# Research on the mechanism of the impact of physical activity on negative emotions of middle school students, and the chain mediating role of social competence and interpersonal relationships

**DOI:** 10.3389/fpsyg.2025.1577987

**Published:** 2025-04-29

**Authors:** Yining Hu, Liangyu Zhao, Wenze Sui, Yan Gao

**Affiliations:** School of Physical Education, Shandong University, Jinan, China

**Keywords:** physical activity, negative emotions, social competence, interpersonal relationships, senior high school students

## Abstract

**Background:**

This study aims to explore the relationship between physical activity and negative emotions (Depression and anxiety) of high school students and to investigate the mechanisms by which social competence and interpersonal relationships play a role.

**Methods:**

A random sampling method was used to select 9,504 students from Shandong Province as the survey subjects. The Physical Activity Questionnaire for Adolescents (PAQ-C), Social Adaptive Behavior Scale (SSBS), Quality of Life Scale for Children and Adolescents (QLSCA), and SCL-90 Symptom Checklist were used to conduct the questionnaire survey.

**Results:**

We found that (1) Physical activity was significantly positively correlated with social competence and interpersonal relationships (*r* = 0.122, 0.182, *p* < 0.01), and significantly negatively correlated with high school students’ negative emotions (*r* = −0.125, *p* < 0.01). Social competence, interpersonal relationships, and negative emotions were significantly negatively correlated (*r* = −0.295 and −0.403, *p* < 0.01); (2) Social competence and interpersonal relationships partially mediate the relationship between physical activity and high school students’ negative emotions, with mediation effect values of −0.138 and −0.445, respectively; (3) Social competence and interpersonal relationships play a chain mediating role in the relationship between physical activity and high school students’ negative emotions, with a mediating effect value of −0.303 and an effect proportion of 25%.

**Conclusion:**

Our results show that physical activity can reduce the level of negative emotions of high school students and enhance their social competence and interpersonal relationships, thus alleviating their negative emotions.

## 1 Introduction

Negative emotions are typically conceptualized as affective states that exert a detrimental effect on an individual’s psychological functioning, characterized by core manifestations including depressed mood, anxiety symptoms, anger reactivity, etc. (Lazarus, 1991; Cheng et al., 2023). Such negative emotional states have emerged as prevalent mental health concerns among adolescent, reflecting not only their psychological adjustment but also being a major cause of illness and disability in this age group (Mental health of adolescents, n.d.). Existing research confirms that depression and anxiety are key components of negative emotions (Muris et al., 2001; Zhang et al., 2019). Alarmingly, China’s youth population is facing a rise in negative emotional challenges. According to the “Annual Evolution of Adolescent Mental Health Status in 2009 and 2020,” the detection rate of depression among Chinese adolescents has increased from 23.2 to 24.6%, and the phenomenon of sleep deprivation continues to worsen. This indicates that nearly one-fifth of adolescents exhibit depressive tendencies, with 17.2% showing mild depressive symptoms and 7.4% meeting the criteria for severe depression (Hao et al., 2023). The high school stage is a critical period for entering adulthood, where students face multiple challenges such as academic pressure, changes in social relationships, and future planning (Yoon et al., 2023; Zhao et al., 2019; Schaffhuser et al., 2017). In addition, high-intensity learning tasks and exam pressure may lead to anxiety, fatigue, and a decrease in self-worth, affecting their future physical and mental health and comprehensive development (Belcher et al., 2021). Therefore, addressing adolescents’ negative emotions and preventing or mitigating their adverse mental health consequences is an important public health objective.

The mental health status of adolescents is influenced by multiple factors, including family background, school education, social environment, and personal lifestyle (Uddin et al., 2024). Notably, sex (in this case, specifically male and female), as an important biosocial variable, may have a differential impact on mental health (Tang et al., 2022). Female adolescents tend to be more prone to negative emotions such as anxiety and depression due to their heightened emotional sensitivity (Tang et al., 2022). Concurrently, physical activity (PA) has gained recognition as a modifiable protective factor against emotional distress. However, with the rapid development of the socio-economy and changes in lifestyle, the level of PA among adolescents is gradually decreasing, with up to 80% of adolescents worldwide failing to reach the recommended level of PA (Dong et al., 2021; Gray et al., 2015). The problems of prolonged sitting and lack of exercise are becoming increasingly prominent, leading to a high prevalence of obesity (Cooper et al., 2015), psychological health issues (such as anxiety and depression) (Makovski et al., 2019), and social behavior issues (de Rezende et al., 2014). It’s worth noting that lower social competence is associated with increased rates of adolescent obesity (Jackson and Cunningham, 2015). Obesity is a key indicator of adolescent physical health; however, the relationship between social competence and mental health status remains to be explored. In addition, poor interpersonal relationships can directly reduce adolescents’ sense of belonging (Renick and Reich, 2021) and may contribute to daytime sleepiness, thereby limiting their opportunities for social activities (Langberg et al., 2016), which in turn increases the risk of developing depression. This is undoubtedly another influential factor that warrants attention. Currently, few studies have explored the mechanisms underlying the relationship between PA and negative emotions among high school students. Therefore, constructing a chain mediation model in this study has significant practical implications for informing and improving mental health education practices for high school students.

As a positive health behavior, PA has unique physiological and psychological benefits (Janssen and Leblanc, 2010). The social psychological mechanism suggests that PA participation can affect individuals’ social psychological cognition and ultimately impact their mental health outcomes (Lubans et al., 2016). In addition, relevant studies have shown that the amount of PA among adolescents has a significant positive impact on their life satisfaction and self-efficacy (Pascoe et al., 2020). Teenagers who underwent 8 weeks of physical confrontation training showed more positive effects on self-esteem and general physical self-concept compared to those who did not participate (Hukkelberg et al., 2019). From this, it can be seen that PA has multiple positive values for the mental health of adolescents. Therefore, we propose hypothesis 1: PA negatively predicts the occurrence of negative emotions.

Social competence refers to an individual’s gradual learning to master social norms, handle interpersonal relationships correctly, self-control, and regulate emotions in order to meet the requirements of the social environment, and effectively adapt to school and social life (Kang et al., 2022). The social competence and development of adolescents are crucial for their future growth and development (Nie et al., 2008). As an important indicator of adolescents’ physical and mental health (Koszałka-Silska et al., 2021), social competence is not only related to academic performance but may also influence the emergence of behavioral problems (Hukkelberg et al., 2019). Therefore, social competence is an important dimension for measuring the level of mental health. Understanding the overall status of social competence can help detect students’ behavioral problems as early as possible and lay the foundation for mental health education (Dirks et al., 2007). According to ecosystem theory, the development process of individuals is embedded in a series of interdependent environmental systems. Students participating in PA in school or community environments enhance their interaction with the surrounding micro-environment, establish positive connections, and promote the synchronous improvement of their social competence and mental health (Zhang and Deng, 2022). Therefore, the study proposes hypothesis 2: Social competence plays a mediating role in the relationship between PA and high school students’ negative emotions.

Interpersonal relationships refer to the psychological distance and emotional bonds that arise between people in the process of interacting with each other, reflecting the psychological state of individuals or groups seeking to satisfy their needs (Luo et al., 2017). Especially during adolescence, which is a stage of social relationship reconstruction, teenagers tend to have specific and novel interpersonal needs, and they need to adapt to new social environments and establish new interpersonal relationships. For high school students, interpersonal relationships include interactions with peers, teachers, parents, and others. The Youth Resilience Model emphasizes the important buffering role of positive interpersonal relationships in the adaptation process of adolescents. For example, positive peer support can help alleviate the negative effects of depression symptoms and peer conflicts in the school environment (Kochel et al., 2017). Therefore, good interpersonal relationships can provide strong emotional support, effectively alleviate psychological problems such as anxiety and depression, and have a promoting effect on adolescent mental health (Chen et al., 2020). On the contrary, poor campus interpersonal relationships are significantly associated with an increase in aggressive behavior and low levels of mental health (Liu et al., 2023). Engaging in PA, especially group activities on campus, provides a platform for interaction and collaboration with peers, which helps to cultivate communication and collaboration skills, and enhance their social interaction abilities (Hosker et al., 2019). Not only is it beneficial for an individual’s social development level, but it also helps establish friendships and social networks (Wilhite et al., 2023). Multiple studies in China have also shown that regular PA can help improve interpersonal relationships and enhance social intimacy between people (Hu and Liu, 2023; Sun and Zhang, 2020). Therefore, the study proposes hypothesis 3: Interpersonal relationships play a partial mediating role in the relationship between PA and high school students’ negative emotions.

Adolescence is a critical period for the formation of psychological, behavioral, and social patterns. Teenagers with low social competence are more likely to be rejected by their peers, thereby reducing interpersonal contact (Koszałka-Silska et al., 2021). In the long run, higher levels of social competence among adolescents can improve their preparation for college, increase their chances of success in their careers, and help establish positive interpersonal relationships, thereby maintaining their mental health (Greenberg et al., 2017). Sports contain rich emotional experiences, moral tempering, and character development. Collective sports activities provide opportunities for interaction and cooperation, which have a positive impact on enhancing children and adolescents’ social competence and promoting social development (Dimitri et al., 2020). Beneficial for the improvement of various positive psychological qualities among adolescents (Shachar et al., 2016). Based on the above analysis, social competence and interpersonal relationships are key mediating factors affecting students’ negative emotions. This study further proposes hypothesis 4: Social competence and interpersonal relationships play a chain mediating effect between PA and high school students’ negative emotions.

**Figure 1:**
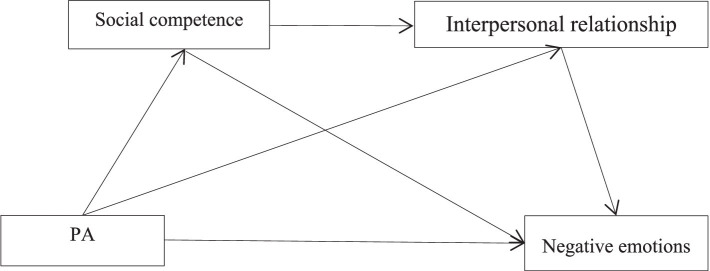
Modelling the mediating effects of social competence and interpersonal relationships.

In summary, this study aims to deeply explore the complex relationship between students’ PA, social competence, interpersonal relationships, and negative emotions, and construct a chain mediation model (as shown in Figure 1). The study will test the following aspects: (1) PA has a significant negative predictive effect on negative emotions; (2) Social competence and interpersonal relationships play an independent mediating role between PA and negative emotions; (3) Social competence and interpersonal relationships play a chain mediating role between PA and negative emotions.

## 2 Materials and methods

### 2.1 Participants

This study was conducted in accordance with the guidelines of the Helsinki Declaration. All procedures involving human subjects have been approved by the Ethics Committee of Shandong University (20180517). Before the investigation began, both parents and students filled out informed consent forms. During the 2020–2021 academic year, data were collected through random sampling from 186 middle and high schools across 17 cities in Shandong Province, China. All staff involved in data collection and processing have received two rounds of standardization training. During the investigation process, trained investigators organized students to use standardized guidelines to measure their physical fitness and guided them to fill out online questionnaires. All data is collected voluntarily, anonymously, and confidentially, and stored on a password-protected website. This study selected data from the 2020–2021 academic year and studied 9,504 randomly selected high school students (age: 16.07 ± 1.26), in Shandong Province. Among them, 4,533 boys (47.70%) and 4,971 girls (52.30%).

### 2.2 Measures

#### 2.2.1 Physical activity

This study adopted the Physical Activity Questionnaire for Adolescents (PAQ-A) developed by foreign scholars (Kowalski et al., 2004). This 9-item instrument assesses adolescents’ PA levels across various contexts using a 5-point Likert scale system. The scale structure is as follows: Item 1 evaluates leisure-time physical activity (e.g., “In the past 7 days, how often did you engage in sports or exercise outside school hours?”). Items 2–7 evaluate PA during physical education (PE) classes lunch breaks, evenings, and weekends (e.g., “How often did you engage in vigorous activities during PE?”). Item 8 captures overall PA frequency across 7 days. Item 9 assesses regular PA habits during the previous week and is excluded from total scoring. The total PA score is calculated as the mean of Items 1–8, with higher scores indicating greater PA levels. The Cronbach’s alpha of the scale in this study was 0.906.

#### 2.2.2 Social competence

This study employed the social competence subscale of the School Social Behavior Scale (SSBS) (Merrell et al., 1993). The original SSBS comprises two primary dimensions—Social Competence and Antisocial Behavior—with the current study focusing exclusively on the social competence dimension. This 32-item subscale evaluates three core aspects of social functioning: academic skills (e.g., “Completing homework in your seat without prodding”), self-management skills (e.g., “Most of the time being able to work with other students”), and interpersonal communication skills (e.g., “Ability to take the initiative to help classmates”). All items were using a 5-point Likert scale. The response options were: 1 = never occurs, 2 = occasionally occurs, 3 = sometimes occurs, 4 = often occurs, and 5 = frequently occurs. The total score ranges from 32 to 160, with higher scores indicating greater social competence. The Cronbach’s alpha of the scale in this study was 0.913.

#### 2.2.3 Interpersonal relationship assessment questionnaire for middle school students

This study used the interpersonal relationships dimensions from the Quality of Life Scale for Children and Adolescents (QLSCA) (Wu et al., 2006), a 49-item instrument comprising 13 dimensions, including teacher-student relationships, peer relationships, parent–child relationships, learning abilities and attitudes, and others. The current study focused on three key interpersonal dimensions: (1) teacher-student relationships (e.g., “How satisfied are you with your relationship with your teachers?”), (2) peer relationships (e.g., “To what extent do you feel accepted by your classmates?”), and (3) parent–child relationships (e.g., “How would you rate your overall happiness with your parental relationships?”). All items were rated using a 4-point Likert scale (1 = never, 2 = occasionally, 3 = often, 4 = always), and the score for each dimension was calculated by summing its component items. The overall interpersonal relationship score was the sum of the three dimension scores. Higher scores indicated stronger interpersonal relationships. The Cronbach’s alpha of the scale in this study was 0.912.

#### 2.2.4 Negative emotions

This study adopted the SCL-90 Symptom Self-Assessment Scale (Chen et al., 2020). The scale has a total of 90 evaluation items. Given that anxiety and depression are core manifestations of negative emotions in middle school students are anxiety and depression, this study has decided to use these two key psychological states as evaluation indicators to measure the negative emotions of middle school students. The measure employs a 5-point Likert scale (1 = Not at all, 2 = A little, 3 = average, 4 = Somewhat, 5 = Very). Representative items from each subscale include: (1) Anxiety: “Feeling nervous or shaky inside.” (2) Depression: “Feeling hopeless about the future.” Subscale scores are calculated by summing relevant items, with higher scores indicating greater symptom severity. The Cronbach’s alpha of the scale in this study was 0.962.

#### 2.2.5 Covariance

We also measured control variables that may affect the results, including sex, age, place of residence, only child status, and parental education level. Sex is represented by two dummy variables (1 = boy, 2 = girl). Residential areas are divided into five categories (1 = the central area of the city, 2 = remote areas of the city, 3 = local township, 4 = other cities, 5 = rural areas). Whether a child is an only child can be divided into two categories (1 = Yes, 2 = No). The educational level of both parents (nine options, no education = 1, primary school = 2, junior high school = 3, vocational school = 4, vocational high school = 5, high school = 6, associate degree = 7, bachelor’s degree = 8, graduate and above = 9).

### 2.3 Data analysis

Establish a database based on the questionnaire content and variables, and use SPSS 26.0 (IBM Corporation, Armonk, NY, United States) (IBM SPSS, 2024) to perform common method bias test, independent sample t-test, descriptive statistics, and correlation analysis on the data. Use SPSS macro program PROCESS 3.5 to conduct mediation effect test.

## 3 Results

### 3.1 Descriptive analysis

Descriptive statistics for each variable are presented in Table 1. A total of 9,504 participants with a mean age of 16.07 years were included in this study. Among them, there were slightly more girls (52.3%) than boys (47.7%). The majority of them lived in the central part of the city and 70.7% were not only children. Most of their parents had completed junior high school education.

**Table 1:** Descriptive statistics of the sample (*N* = 9,504).

Variables	Definition	Frequency	Percentage (%)	Mean	SD
Sex	Boys	4,533	47.7	1.48	0.50
	Girls	4,971	52.3		
Residence	The central area of the city	4,176	43.9	2.56	1.62
	Remote areas of the city	703	7.4		
	Local township	2068	21.8		
	Other cities	281	3		
	Rural areas	2,276	23.9		
Only child status	Yes	2,787	29.3	1.29	0.46
	No	6,717	70.7		
Father’s education	No education at all	182	1.9	4.34	1.98
	Primary school	915	9.6		
	Junior high school	3,889	40.9		
	Secondary/technical school	917	9.6		
	Vocational high school	436	4.6		
	General high school	1,446	15.2		
	University college	819	8.6		
	Undergraduate	751	7.9		
	Postgraduate and above	149	1.6		
Mother’s education	No education at all	391	4.1	3.94	2.00
	Primary school	1744	18.4		
	Junior high school	3,606	37.9		
	Secondary/technical school	864	9.1		
	Vocational high school	354	3.7		
	General high school	1,156	12.2		
	University college	658	6.9		
	Undergraduate	591	6.2		
	Postgraduate and above	140	1.5		
Age	Continuous variable	9,504		16.07	1.26
PA	Continuous variable	9,504		3.98	1.35
Negative emotions	Continuous variable	9,504		35.75	15.22
Social competence	Continuous variable	9,504		122.02	24.81
Interpersonal relationships	Continuous variable	9,504		42.61	7.70

PA, Physical activity.

### 3.2 Common method deviation testing

Harman single factor analysis was used to perform a common method bias test on the data. The results showed that there were 7 factors with eigenvalues greater than 1, and the variance explained by the first factor was 32.761% (less than 40%), indicating that there was no significant common method bias in this study.

### 3.3 Sex differences in PA, social competence, interpersonal relationships and negative emotions

Independent sample t-test was used to compare the differences in PA, social competence, interpersonal relationships, and negative emotions between boys and girls. As shown in Table 2, the PA scores of boys are higher than those of girls, but the total scores of social competence, interpersonal relationships, and negative emotions are significantly lower than those of girls.

**Table 2:** Analysis of sex differences in PA, social competence, interpersonal relationships and negative emotions (*N* = 9,504).

Variable	Sex (mean±SD)		*t*	*p*
	Boys (*N* = 4,533)	Girls (*N* = 4,971)		
PA	4.27 ± 1.48	3.71 ± 1.16	20.29	0.000**
Social competence	120.34 ± 26.94	123.56 ± 22.58	−6.273	0.000**
Interpersonal relationships	42.29 ± 8.13	42.90 ± 7.29	−3.792	0.000**
Negative emotions	34.42 ± 14.90	36.96 ± 15.42	−8.16	0.000**

* *p* < 0.05, ** *p* < 0.01. PA, Physical activity.

### 3.4 Correlation analysis of PA, interpersonal relationships, social competence, and negative emotions

We used the Pearson correlation coefficient of SPSS 26.0 to conduct correlation analysis on the main variables. The test results are shown in Table 3. The results showed that PA was negatively correlated with negative emotions (*r* = −0.125, *p* < 0.01), and significantly positively correlated with social competence (*r* = 0.122, *p* < 0.01) and interpersonal relationships (*r* = 0.182, *p* < 0.01); Social competence is significantly positively correlated with interpersonal relationships (*r* = 0.595, *p* < 0.01), while social competence, interpersonal relationships, and negative emotions are negatively correlated (*r* = −0.295, *p* < 0.01) (*r* = −0.403, *p* < 0.01). The results of the test showed that with the increase in PA, social competence and better interpersonal relationships of the adolescents, fewer negative emotions appeared.

**Table 3:** Correlation analysis of PA, interpersonal relationships, social competence and negative emotions.

Variable	PA	Social competence	Interpersonal relationship	Negative emotions
PA	1			
Social competence	0.122**	1		
Interpersonal relationship	0.182**	0.595**	1	
Negative emotions	−0.125**	−0.295**	−0.403**	1

* *p* < 0.05, ** *p* < 0.01. PA, Physical activity.

### 3.5 Chain mediation effect analysis of PA, interpersonal relationships, social competence, and negative emotions

Regression analysis was conducted with negative emotions as the dependent variable, adolescent PA as the independent variable, and social competence and interpersonal relationships as mediating variables, while controlling for factors such as sex, place of residence, only child status, and parental education level. The regression analysis results after controlling for variables showed that PA significantly negatively predicted negative emotions (*β* = −1.193, *p* < 0.01). At the same time, we also found that PA can significantly positively predict social competence (β = 2.512, *p* < 0.01) and interpersonal relationships (β = 0.654, *p* < 0.01). Social competence can positively predict interpersonal relationships (β = 0.177, *p* < 0.01). Social competence and interpersonal relationships negatively predict negative emotions (β = −0.055, *p* < 0.01) (β = −0.680, *p* < 0.01). These results are shown in Table 4.

**Table 4:** The mediating effect of social competence, interpersonal relationships.

Variable	Negative emotions		Social competence		Interpersonal relationship		Negative emotions	
	*β*	*sx*	*β*	*sx*	*β*	*sx*	*β*	*sx*
Sex	1.768**	0.32	4.061**	0.516	0.348**	0.131	2.718**	0.294
Residence	−0.112	0.103	0.622**	0.167	0.008	0.042	0.003	0.095
Only child	−0.07	0.359	4.918**	0.58	0.549**	0.147	1.167**	0.331
Father’s level of education	−0.409**	0.103	1.044**	0.167	0.149**	0.042	−0.124	0.095
Mother’s level of education	−0.488**	0.105	1.002**	0.169	0.095*	0.043	−0.247*	0.096
PA	−1.193**	0.117	2.512**	0.188	0.654**	0.048	−0.306**	0.109
Social competence					0.177**	0.003	−0.055**	0.007
Interpersonal relationship							−0.680**	0.023
*R^2^*	0.029		0.046		0.37		0.182	
*F*	75.902**		797.593**		48.021**		264.694**	

* *p* < 0.05, ** *p* < 0.01. PA, Physical activity.

**Figure 2:**
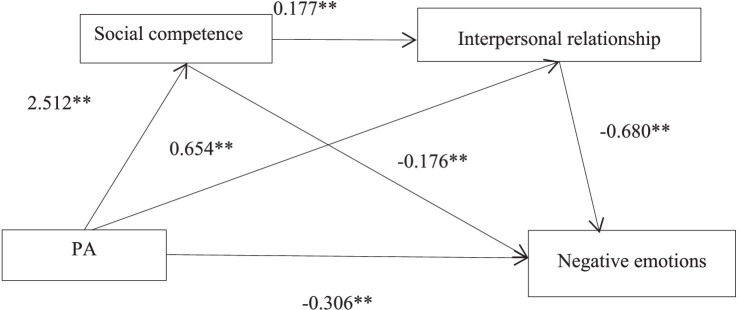
The mediating effect of social competence, interpersonal relationships.

The bootstrap method was used to test the mediation effect, and the results showed that three paths were significant (see Table 5 and Figure 2). In the path of “PA → social competence → negative emotions,” the indirect effect was −0.138, 95% CI [−0.210, −0.075], accounting for 12% of the total effect. In the path “PA → interpersonal relationship → negative emotions,” the indirect effect was −0.445, 95% CI [−0.562, −0.341], accounting for 37% of the total effect. The 95% confidence intervals of bootstrap did not contain 0, indicating that the mediating roles of social competence and interpersonal relationship were both significant in the influence of adolescents’ PA on negative emotion. For the chain mediation path “PA → social competence → interpersonal relationships → negative emotions,” the indirect effect value was −0.303, 95% CI [−0.412, −0.215], accounting for 25% of the total effect. The 95% bootstrap confidence interval also did not contain 0. This indicates that social competence and interpersonal relationships play a chain mediating role in the relationship between PA and negative emotions among adolescents, supporting the validity of all research hypotheses. This suggests that adolescents enhance their social competence by engaging in PA, which in turn enhances interpersonal relationships and ultimately reduces negative emotions among high school students.

**Table 5:** Bootstrap test results and effect decomposition of mediated effects.

Effect	trails	Effect	SE	LLCI	ULCI	Efficiency ratio (%)
Aggregate effect	PA → Negative emotions	−1.193	0.117	−1.421	−0.964	100.00%
Direct effect	PA → Negative emotions	−0.306	0.109	−0.52	−0.092	25.60%
Total indirect effect		−0.887	0.006	−0.089	−0.068	74.30%
Indirect effects process	PA → Social competence →Negative emotions	−0.138	0.002	−0.017	−0.008	11.60%
	PA → Interpersonal relationship →Negative emotions	−0.445	0.004	−0.048	−0.031	37.30%
	PA → Social competence →Interpersonal relationship →Negative emotions	−0.303	0.003	−0.034	−0.021	25.40%

PA, Physical activity.

## 4 Discussion

This study established a chain mediation model to explore how PA affects the negative emotions of adolescents. The results showed that social competence and interpersonal relationships partially mediate the relationship between PA and high school students’ negative emotions, while social competence and interpersonal relationships play a chain mediated role in the relationship between PA and high school students’ negative emotions. This provides a new understanding of guiding and optimizing negative emotions education practices for students.

### 4.1 Analysis of sex differences

The study first observed significant gender differences: high school boys generally exhibit higher levels of PA than girls, which is consistent with previous research findings. Longitudinal studies have shown that PA levels are consistently higher among high school boys than girls, and that PA intensity declines 2.3 times faster among females than males during puberty—a pattern closely associated with physiological changes in females, body imagery anxiety, and societal gender role expectation (Armstrong and Welsman, 2006; Halliday et al., 2019). International research has found that with the onset of puberty, the overall PA of adolescents tends to decline, and in most countries and regions around the world, girls have significantly fewer opportunities to participate in physical exercise and other physical activities than boys (van Sluijs et al., 2021). The underlying cause is structural inequality: only 19 percent of PA programs offered in schools and communities cater to girls’ preferred non-competitive, socially oriented activities, while 76 percent are dominated by competitive team sports favored by boys, resulting in a relative lack of options aligned with girls’ specific needs and interests (McMahon et al., 2017). At the same time, we found that girls tend to exhibit stronger social skills and interpersonal relationships but are more prone to negative emotions compared to boys. Some research results indicate that compared to boys, girls tend to exhibit stronger social competence and better interpersonal communication abilities and show milder behavioral problems (Hukkelberg et al., 2019). Evidence shows that boys with more sensitive or introverted personalities are more likely to feel lonely and lack confidence in their social competence than girls (Perron-Gélinas et al., 2017). In addition, girls prefer to maintain a small but close social circle of friends, which is an important source of emotional support for them (Li et al., 2023). It is worth noting that, despite objectively stronger social competence, girls report higher levels of negative affect compared to boys. Previous studies have shown that girls’ overall mental health may be poorer than that of boys (Yoon et al., 2023; Campbell et al., 2021), and that the sex difference peaks during adolescence (Salk et al., 2017). This may stem from the inherent differences in emotional expression between the sexes, with girls exhibiting greater emotional empathy and self-reflection (Li et al., 2018), but lower self-efficacy and emotional resilience (Tomyn and Weinberg, 2016). Additionally, the early onset of puberty may make girls more susceptible to emotional distress (Yoon et al., 2023). However, if girls can develop good problem-solving strategies in late adolescence and receive support from their families and peers, their risk of experiencing mental health problems will be greatly reduced (Moore et al., 2018).

### 4.2 Relationship between physical activity and negative emotions

Our research findings reveal a significant negative correlation between PA and negative emotions: the higher the level of student participation in PA, the lower the likelihood of exhibiting symptoms of depression and anxiety. This discovery further confirms the conclusions of earlier research, the positive impact of PA on negative emotions is multidimensional. Previous studies have shown that exercise and fitness can enhance the self-regulation ability of the adolescent brain and improve individual psychological resilience (Belcher et al., 2021). When facing high-intensity learning tasks and personal life challenges, PA has become an effective way to alleviate stress caused by academic pressure, interpersonal relationships, and other issues, helping to reduce the occurrence of anxiety and depression and maintain a good mental health state. In particular, group activities such as running, team sports, and yoga have been shown to significantly reduce loneliness and depression symptoms (Rodriguez-Ayllon et al., 2019). In addition, aerobic exercise and moderate intensity physical training make important contributions to emotional management (Costigan et al., 2016). These types of exercises may even bring a significant increase in happiness (McMahon et al., 2017). Therefore, there is a significant negative correlation between PA and students’ negative emotions such as anxiety and depression. Encouraging and promoting students to actively participate in various PA is not only beneficial for their physical health but also an important means to ensure the mental health of adolescents and promote their comprehensive growth.

### 4.3 The mediating role of social competence and interpersonal relationships

Social competence was found to partially mediate the relationship between PA and negative emotions. This is consistent with previous research findings. PA is considered a key factor in promoting the physical and mental health development of adolescents, and their role in enhancing social competence has been fully recognized (Okely et al., 2022). Domestic research also points out that the participation of teenagers in PA has a positive impact on the development of their social competence, which can effectively enhance their social interaction and ability to handle affairs (Sun et al., 2019; Zhu and Shu, 2022). At the same time, the stronger a student’s social competence, the better their mental health (Tang et al., 2021). There are also studies internationally showing that intervening in sports can effectively improve teenagers’ self-control ability and help stimulate more positive emotions (Shachar et al., 2016). Sports activities, as a collective practical activity, provide a natural social environment for adolescents. In team sports, they must learn to communicate and collaborate with teammates, abide by rules, respect opponents, and handle the relationship between competition and cooperation. These experiences help improve their ability to adapt to social needs in different situations (Weiss, 2020). To better cope with various pressures and challenges in daily life and establish a positive attitude towards life.

In addition, we have found that PA can improve negative emotions by enhancing interpersonal relationships. Previous studies have shown that interpersonal networks are an essential factor in ensuring individual mental health (Lamblin et al., 2017). This is because teenagers’ skills in handling interpersonal relationships may not be mature enough, and when individuals have interpersonal problems, it can lead to conflicts and feelings of loneliness, often closely related to low levels of mental health (Lee et al., 2021). In addition, studies have found that different sources of social support have varying effects on the mental health status of adolescents. The alienation of parent–child relationships can make teenagers more prone to developing a tendency towards self-doubt, making it difficult for them to adapt to and cope with the complexity and diversity of learning and life (Sheeber et al., 2007). Poor campus interpersonal relationships, social isolation, and social difficulties may lead to damaged self-esteem, and decreased self-confidence, and even trigger psychological problems such as social phobia, causing individuals to feel lonely and excluded (Ge and Zhang, 2024). However, teenagers who actively engage in PA every week show a significant positive correlation with their classmates (Quan and Lu, 2020). The social bonds formed by regularly participating in PA are considered to have a positive effect on negative emotions (Monshouwer et al., 2013). Another study showed that after a 4 week exercise intervention, adolescents showed improvements in social competence, physical function, body image, and overall self-worth, and changes in social relationships predicted improvements in negative emotions (Ullrich-French et al., 2012). Our research further demonstrates that by enhancing support for intimate relationships, PA can help alleviate the negative impact of potential psychological problems. Therefore, actively advocating for teenagers to participate in team sports activities can undoubtedly effectively broaden their social network and gain profound emotional satisfaction (Beenen et al., 2023). This is crucial for maintaining mental health.

### 4.4 Chain mediation effect

Finally, the study found that social competence and interpersonal relationships play a chain mediating role in the relationship between PA and negative emotions. Teenagers who are able to successfully adapt to the school environment are more likely to develop positive interpersonal interaction patterns, which directly affect their interpersonal behavior (Chang et al., 2020). Engaging in PA among teenagers helps to develop a sense of rules and cooperation, shaping good sportsmanship such as teamwork and social cooperation (Yan et al., 2020). This indirectly enhanced their social competence. And good social competence, as an important component of social adaptation, directly affect an individual’s social competence and social outcomes (Twenge et al., 2007). Human beings are social animals, and participating in PA inevitably increases their interaction with others. However, having good interpersonal skills can win better teacher-student and classmate relationships (Lynn et al., 2003). When teenagers encounter difficulties, the understanding and support of friends and teachers can effectively reduce their psychological burden, prevent and alleviate negative emotions. In addition, participating in PA with family members in a family environment can not only enhance personal psychological resilience, but also deepen the bond between parents and children. Therefore, PA, with their unique educational value and practical significance, not only shape students’ strong physical fitness, but also comprehensively cultivate students’ adaptation to school life, establish good interpersonal relationships, and greatly promote their mental health growth.

### 4.5 Limitation

This study explored the relationship between PA and negative emotions among high school students, constructed a chain mediation model, reveals the underlying mechanism of the impact of PA on negative emotions among high school students, and provided preliminary evidence for exploring the causal relationship between these variables. There are also some shortcomings in this study. Firstly, this study is a cross-sectional study and cannot prove the exact causal relationship between the variables. In addition, the data for this study were collected through self-reports, PA was assessed using a self-reported questionnaire. Relying solely on this method may have introduced recall bias and other issues that could affect the study’s results. Therefore, future studies could employ longitudinal data to examine variable interactions and better understand their causal relationships. Methodologically, future research could consider mixed methods (combining quantitative and qualitative approaches) to more fully capture the complexity of the factors involved. Simultaneously using multiple sources of information to collect data to improve the reliability of research results.

## 5 Conclusion

In summary, this study systematically reveals the multilevel mechanism through which physical activity alleviates negative emotions in adolescents by constructing a chain mediation model. It was found that physical activity not only alleviates negative emotions through the two independent paths of enhancing social competence and improving interpersonal relationships, but more importantly, it plays a sustained role in promoting negative emotions through the chain mediation path of “Physical Activity → Social Competence → Interpersonal Relationships → Negative Emotions.” These findings have far-reaching implications for promoting adolescents’ healthy development. Encouraging high school students to actively participate in physical activities—whether team sports, individual exercise, or leisure pursuits—can help to promote their mental health and overall well-being. Enhancing physical fitness while developing adolescents’ social adaptability and interpersonal skills creates a virtuous cycle that supports mental health. These findings provide an important theoretical foundation and practical guidance for developing adolescent health promotion programs that address the multi-dimensional benefits of physical, psychological, and social well-being. Future research could further explore the varied manifestations of psychosocial benefits and the scope of their effects under different types of physical activity.

## Data Availability

Publicly available datasets were analyzed in this study. This data can be found at: Database of Youth Health (https://www.ncmi.cn/index.html).
